# Impact of living arrangements and internet use on the mental health of Chinese older adults

**DOI:** 10.3389/fpubh.2024.1395181

**Published:** 2024-12-06

**Authors:** Ruyu Zhong, Wenwen Ning

**Affiliations:** ^1^School of Sociology, Wuhan University, Wuhan, China; ^2^School of Law and Economics, Wuhan University of Science and Technology, Wuhan, China

**Keywords:** living arrangements, internet use, mental health, older adults, China

## Abstract

**Introduction:**

The consequences of aged living arrangements on mental health in the digital age have drawn significant research attention.

**Methods:**

This study used empirical data to analyze the impact of living arrangements on the mental health of older adults by ordinary least squares (OLS) and to examine the moderating effect of Internet use in it through the moderating effect test. A total of 17,243 older adults were included in the analytical model.

**Results:**

We found that living independently has a negative impact on the mental health of older adults and Internet use can improve the mental health of older adults. There are moderating mechanisms of Internet use in the impact of living arrangements on the mental health of older adults, but it is necessary to look at the moderating mechanisms of different patterns of Internet use. Using the Internet for social interaction (chatting and information acquisition) can weaken the impact of living arrangements on the mental health of older adults, while unidirectional Internet use (entertainment and financial management) strengthens the impact of living arrangements on the mental health of older adults.

**Disscusion:**

Therefore, this study puts forward the following suggestions: first, to develop family care for older adults and pay attention to the positive role of intergenerational support in the mental comfort of older adults; second, it is imperative for the government and social service departments to assist older adults in establishing correct concepts of Internet use, enhancing their digital literacy, and improving their digital skills.

## 1 Introduction

The global population aging trend is accelerating, and China’s population is also aging at unprecedented levels. By the end of 2021, the number of people over 60 years old in China is as high as 260 million, accounting for 18.9% of the population of the country. Due to the increasing number of older adults, many issues related to it have gradually emerged and attracted the attention of the whole society. Among them, mental health is one of the hot topics (1). As a positive phenomenon (2), mental health is directly related to the quality of life for older adults. Therefore, it is crucial to fully recognize the importance of mental health for older adults (3).

As an important factor affecting the mental health of older adults, living arrangements have attracted much attention from scholars. Most research indicates that there is a close relationship between living arrangements and mental health among older adults (4–6); living arrangements have a significant effect on the risk of depression in older adults (7–9). There are two main views on the impact of living arrangements on the mental health of older adults. One view is that living with children is more beneficial to the mental health of older adults than living alone or only with a spouse. Studies have found that living with children is positively associated with the mental health of older adults (10). A contrary view is that living alone or only with a spouse is more favorable to the mental health of older adults. This is because living with children may lead to an over-reliance on their children’s support, which may increase their sense of worthlessness and lead to cognitive impairment (11). Meanwhile, living with children may also lead to a high level of intergenerational conflict (12), which is not conducive to the mental health of older adults. At the same time, scholars have begun to pay attention to the ways in which living arrangements affect the mental health of older adults. Xu (13) found that the influence of living arrangements on the mental health of older adults depends on the sex of children who live with older adults and the living distance between children and their parents. Hamid et al. (14) further found that the interaction between living arrangements and social networks has a significant impact on the mental health of older adults. Regardless of living arrangements, older adults with strong social networks have better mental health than those who live alone and have fewer social networks. Although the relationship between living arrangements and the mental health of older adults has been discussed, the difference in the impact of different living arrangements on the mental health of older adults still needs further verification.

With the continuous increase in Internet use and the increasing popularity of mobile smartphones, more and more older adults use the Internet for shopping, traveling, social communication, and so on. Internet use is also silently having an impact on the mental health of older adults and has been widely discussed by scholars (15–17). On the one hand, research has found that Internet use has a positive impact on the mental health of older adults (18). While increasing older adults’ self-efficacy (19), Internet use can also improve their loneliness (20–22), reduce their depression (23, 24), and decrease their mental distress (25). On the other hand, research has also found that Internet use can negatively affect the mental health of older adults. Using 2014 and 2016 CLASS data, Xie et al. (26) found that Internet use has a negative impact on the mental health of older adults and increases the incidence of depression in older adults.

The above studies mostly investigated the impact of living arrangements or Internet use alone on the mental health of older adults and rarely discussed the influence of both factors on mental health. Ecological system theory (27) holds that many factors related to individuals and the interaction between them will affect people’s mental health. Therefore, based on the current Internet era, while considering the impact of living arrangements on the mental health of older adults, it is also necessary to include Internet use as a factor in order to better improve the mental health of older adults.

In summary, this study proposes the following hypotheses:

*Hypothesis 1*: Older adults living independently have significantly poorer mental health than those who live with their children.

*Hypothesis 2*: Older adults using the Internet have significantly better mental health than those who not using the Internet.

*Hypothesis 2.1*: Older adults with available Internet connections have better mental health than those without available Internet connections.

*Hypothesis 2.2*: The more frequently older adults use the Internet, the better their mental health.

*Hypothesis 2.3*: Older adults who use the Internet for chatting, information acquisition, entertainment, or financial management have better mental health.

*Hypothesis 3*: Internet use can modify the impact of living arrangements on the mental health of older adults by attenuating the negative effects of living independently.

## 2 Materials and methods

### 2.1 Data

The data in this paper come from the 2018 China Health and Retirement Longitudinal Study (CHARLS), which covers families and individuals aged 45 years and above for 150 county-level units and 450 village-level units across the country; 19,816 valid cases are fairly representative, especially for those in the older adult group. It covers basic personal information, physical health and function, psychological status, participation in basic endowment insurance and basic medical insurance, working situation, income status, and basic family information. This source provides detailed data to support the study of issues such as retirement. At the same time, these data also cover related to Internet use. Therefore, it can be applied to the study of the relationship between living arrangements, Internet use, and mental health for older adults. This paper defined older adults as those over 60 years old. Based on age, the sample selected in this paper was the population over 60 years old. After eliminating the missing and invalid variables, 17,243 valid cases were obtained.

### 2.2 Measurement

#### 2.2.1 Dependent variable

The dependent variable in this study was the mental health of older adults. We used the Depression Index to measure mental health, measured by the CESD-10 (Center for Epidemiological Studies Depression Scale) in the CHARLS data (28). This scale contained 10 questions measuring respondents’ mental health and depression levels, with a minimum score of 0 and a maximum score of 30. In the analysis presented here, scores above 10 were considered to be prone to depression, with higher scores indicating greater depression proneness and poorer mental health.

In this study, subjective life satisfaction was utilized as an alternative dependent variable to evaluate the robustness of the research outcomes. Consistent with prior scholarly endeavors, a statistically significant correlation has been established between the depression index (13) and subjective life satisfaction (29), which has been previously employed as a proxy variable for robustness assessment in certain research contexts. Our initial analysis further substantiates a robust correlation between these two constructs (correlation coefficient: −0.4536, achieving statistical significance at the 0.001 level). Therefore, we adopted subjective life satisfaction as an alternative dependent variable in our analysis. We utilized the item from the questionnaire: “Please think about your life-as-a-whole. How satisfied are you with it?” for measurement purposes. This is a five-category ordinal measurement. In this paper, responses in categories 1–3 were consolidated and treated as “satisfied,” while those in categories 4 and 5 were merged as “dissatisfied,” assigned with values of 1 and 0, respectively, resulting in a dichotomous dummy variable.

#### 2.2.2 Independent variable

The independent variable was the living arrangements of older adults. Through the preliminary analysis, it was found that except for living alone, living with a spouse, and living with children, other types of cohabiting family structures only account for a small number. In addition, this paper focused on the intergenerational relationships associated with living arrangements. Therefore, this paper divided living arrangements into two categories: living independently (including living alone or living with a spouse only) (30) and living with children, and treated the other few categories as missing.

#### 2.2.3 Moderating variable

The moderating variable was Internet use, which was users’ behavior in terms of accessing Internet services through various terminal devices (including computers, tablets, and mobile phones). In a questionnaire survey, responses were elicited by asking the question, “Have you used the Internet in the last month.” At the same time, Internet use was divided into three dimensions: available Internet connections, frequency of Internet use, and patterns of Internet use. Available Internet connections were measured according to whether the relevant terminal equipment is owned and whether the current housing has broadband access. The frequency of Internet use was examined by asking the question “What was the frequency of Internet access in the past month,” which was treated as an interval-scale variable. Patterns of Internet use included chatting, information acquisition, entertainment, and financial management. The measurement is based on the question in the questionnaire, “What do you usually do on the Internet?” Answer 1 represents “chatting,” and Answer 2 is “reading news,” which we use to measure the functional use of information acquisition. Answer 5 is “financial management,” which is used to measure the use of online financial functions. The remaining answers, “3. Watch videos” and “4. Play games,” were consolidated and named “entertainment,” while Answer “6. Others” was treated as missing. Based on this, a four-category variable was obtained and further processed into four binary dummy variables, each with values of 1 (“yes”) and 0 (“no”).

#### 2.2.4 Control variables

Personal characteristics (sex, age, marital status, and physical health) and social structural characteristics (education level, total income, and place of residence) were included as control variables (Table 1).

**Table 1:** Definition of key variables and descriptive analysis (*N* = 17,243).

Variable		Definition	Mean	Std. dev.
Dependent Variable	Mental health	Measuring the level of depression index based on the depression scale CES-D(10):[0,30]	7.633	5.783
	Subjective Life satisfaction	The substitute variable of the robustness test, whether satisfied with life: 1 = satisfied, 0 = dissatisfied	0.926	0.262
Independent Variable	Living arrangements	1 = living alone or living with a spouse only, 0 = living with children	0.419	0.493
Moderating variable	Internet use	Whether older adults use the Internet: 1 = yes, 0 = no	0.073	0.261
	Available Internet connections	Whether there is an Internet connection at home: 1 = Yes, 0 = No	0.073	0.261
	Frequency of Internet use	Frequency of Internet use in the last month: [1,4], never, occasionally, almost every week, almost daily	1.205	0.744
	Chatting	Whether you can use the Internet for chatting: 1 = yes, 0 = no	0.044	0.205
	Entertainment	Whether you can use the Internet for entertainment: 1 = yes, 0 = no	0.055	0.227
	Information acquisition	Whether you can use the Internet to get information: 1 = yes, 0 = no	0.061	0.239
	Financial management	Whether you can use the Internet for financial management: 1 = yes, 0 = no	0.005	0.07
Personal characteristics	Age	Actual age	68.799	6.363
	Sex	Sex of older adults: 1 = male, 0 = female	0.482	0.5
	Marital status	Whether has a spouse living together: 1 = yes, 0 = no	0.894	0.308
	Physical health	Whether older adults are healthy: 1 = healthy, 0 = unhealthy	0.218	0.413
Social structural characteristics	Education level	The educational level of older adults: [1,4], illiterate, elementary school and below (literate), middle school, high school, and above	2.075	0.966
	Total income	Total number of income in a year (yuan)	10914.685	20376.547
	Place of residence	Place of residence: 1 = urban, 0 = rural	0.271	0.445

### 2.3 Analysis strategy

#### 2.3.1 OLS model

In this paper, the method of ordinary least squares (OLS) regression was used to estimate the impact of living arrangements on the mental health of older adults:


(1) $${\mathrm{Depression}}_{i}=\alpha +\beta •{\mathrm{Living}}_{i}+\gamma •X_{i}+\varepsilon_{i}$$

In Equation 1: *Depression_i_* is the value of the depression index of the *i* th older adult; *Living_i_* is the living arrangement of the *i* th older adult; *Xi* represents the personal and social structural characteristics of the *i* th older adult.

#### 2.3.2 Robust test: PSM and alternative dependent variable

As the coefficient estimated by OLS regression may be affected by selectivity bias and reverse causality, this paper adopted the PSM (propensity score matching) method as a robustness test for OLS estimation. First, we utilized the logit model to predict the probability of individuals not living with their children, referred to as the propensity score. In this process, the controlled covariates *X_i_* were the same as the control variables in the OLS model. Second, we matched individuals whose propensity scores were within a common range of values. In order to ensure the robustness of the results, we used four matching methods: nearest neighbor matching (*k* = 1), nearest neighbor matching within a radius, kernel matching, and local linear regression matching. Then, the sample balance test was carried out. Finally, we calculated the “average treatment effect on the treated” (ATT) from the matched samples.

To further test the reliability of the findings, this paper utilized life satisfaction from CHARLS (2018) as the explained variable to measure the mental health of older adults and repeated the empirical analysis process of the main model.

#### 2.3.3 Heterogeneity analysis and chow test

In order to more carefully examine the differences in the study results between the different groups, the heterogeneity test was conducted by group regression. At the same time, the Chow test was used to test the significance of the difference between groups.

#### 2.3.4 Moderating effect

In order to examine the moderating effect of Internet use, this paper used the moderating effect test, and this model was set up as follows:


(2) $$\begin{array}{l} De\mathrm{pressio}n_{i}=\alpha_{2}+\beta_{2}•\mathrm{Livin}g_{i}+\gamma_{2}•X_{i}+\delta •\mathrm{Interne}t_{i}+\\ \phi •\mathrm{Livin}g_{i}\times \mathrm{Interne}t_{i}+\varepsilon_{2} \end{array}$$

(3) Depressioni=α3+β3•Livingi+γ2•Xi+∑k=16δk•Internetki'+∑k=16ϕk•Livingi×Internetki'+ε3

In Equation 2: *Internet_i_* is the moderating variable, which is Internet use of older adults; and the rest of the variables and symbols have the same meaning as in Equation 1.

In Equation 3: To further investigate how Internet use plays a moderating role, this paper used behavioral characteristics of Internet use (availability of devices, frequency of Internet use, and patterns of Internet use) to analyze the mechanism of Internet use in the impact of living arrangements on the mental health of older adults.

## 3 Results

### 3.1 Empirical results and analysis

For the analysis of the effect of living arrangements on mental health, this study used OLS Regression. Model 1 includes independent variables only to build the benchmark model. Model 2 adds all the control variables to Model 1, and Model 3 adds Internet use to Model 2. Model 4 replaces Internet use by using available Internet connections, frequency of Internet use, and patterns of Internet use and examines the specific impact of Internet use from three dimensions.

Table 2 shows that living arrangements have a significant impact on the mental health status of older adults (Model 1 and Model 2). Under the condition of controlling personal characteristic variables and regional characteristic variables, the estimation results show that the level of depression index for older adults who live not with children is significantly higher than that for older adults who live with children (*p* < 0.01). At the same time, it can be seen that personal information variables and regional affiliation variables have a significant impact on mental health status. The level of depression is significantly higher among older adults who are male, single, unhealthy, poorly educated, with low income, and living in rural areas. Hypothesis 1 is verified.

**Table 2:** Results of OLS regression analysis for living arrangements, internet use, and mental health of older adults.

	Model 1	Model 2	Model 3	Model 4
Living arrangements	0.340***	0.445***	0.453***	0.455***
	(0.089)	(0.085)	(0.085)	(0.085)
Internet use			−0.888***	
			(0.174)	
Available Internet connections				−1.126
				(0.868)
Frequency of Internet use				0.283
				(0.284)
Chatting				−0.210
				(0.315)
Entertainment				0.200
				(0.355)
Information acquisition				−0.799*
				(0.411)
Financial management				1.369**
				(0.607)
Control variable	No	Yes	Yes	Yes
Constant	7.490***	10.296**	10.626**	10.357**
	(0.058)	(4.192)	(4.189)	(4.196)
*N*	17,243	17,243	17,243	17,243
Adj. R^2^	0.0008	0.1278	0.1290	0.1293

(1) Standard errors in parentheses. (2) * *p* < 0.1, ** *p* < 0.05, *** *p* < 0.01.

Model 3 shows that Internet use significantly reduces the depression index of older adults. Compared with older adults who do not use the internet, older adults who use the Internet do have a lower level of depression index (*p* < 0.01). Hypothesis 2 is verified.

Model 4 indicates that the three dimensions of Internet use have different effects on the mental health of older adults. Available Internet connections and frequency of Internet use both do not have a significant impact on the mental health of older adults (*p* > 0.1). Hypothesis 2.1 and Hypothesis 2.2 are rejected. From the regression coefficient, available Internet connections can reduce the level of depression in older adults, but the higher the frequency of Internet use, the higher the level of depression in older adults. In addition, different Internet use patterns also have different effects on the mental health of older adults. Using the information acquisition function can significantly reduce depression levels in older adults (*p* < 0.1). However older adults who use the financial management function are more depressed than those who do not use this function (*p* < 0.05). The other two patterns of Internet use (chatting and entertainment) have no significant effect on the mental health of older adults (*p* > 0.1). Hypothesis 2.3 is partly verified and partly rejected, indicating the intricate nature of the mechanisms associated with Internet use.

### 3.2 Robustness test

#### 3.2.1 PSM

As the living arrangements of older adults are a self-selecting behavior, there are significant differences between the group living independently (treatment group) and the group living with children (control group) in many aspects, such as age, sex, marital status, education level, and urban–rural differences. Although the OLS model controls these confounding variables, it is difficult to solve the self-selection problems effectively. Therefore, the PSM method is introduced to further validate the robustness of the model.

**Figure 1:**
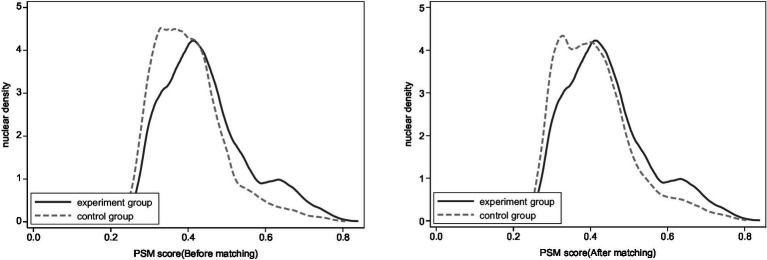
Changes in nuclear density before and after matching.

Table 3 shows the average treatment effect on the treated (ATT) of living arrangements on the mental health of older adults. The results show that the average treatment effects obtained through these four matching methods are significant at the 1% level. The matching outcomes indicate that, after eliminating sample biases between the control and the treatment groups, living arrangements still have a significant impact on the mental health of older adults. Additionally, it can be observed that the average treatment effect on the treated (ATT) derived from neighbor (4) matching, radius matching, and kernel matching are relatively close, which corroborates the robustness of the previous research findings and further confirms Hypothesis 1. Figure 1 shows the changes in kernel density plots before and after matching. From the distribution of nuclear density of PSM score in different groups, it can be seen that the difference between the treatment group and the control group was smaller after matching compared to before matching. In general, after controlling for the influence of confounding variables in the sample, living arrangements still have a genuine and significant effect on the mental health of older adults.

**Table 3:** Results of propensity score matching estimation of living arrangements on the mental health of older adults.

	Treated	Controls	ATT	S.E.	T-stat
Kernel	7.833	7.412	0.421	0.093	4.51***
Neighbor (4)	7.833	7.519	0.314	0.111	2.83***
Radius	7.833	7.410	0.423	0.093	4.54***
Llr	7.833	7.481	0.353	0.124	2.84***

#### 3.2.2 Substituting dependent variable

This paper used a replacement model and a replacement variable to verify such research results. We replaced the dependent variable with life satisfaction and treated it as a binary “dummy” variable that was assigned a value of 0 for dissatisfaction and a value of 1 for satisfaction. Then, logit regression was used to test the effect of living arrangements and Internet use on life satisfaction (Model 5, Model 6, and Model 7).

Table 4 shows that the effects of both living arrangements (Model 5) and Internet use (Model 6 and Model 7) on life satisfaction were statistically significant, which is consistent with the baseline regression results. This supports our conclusion that older adults living independently have significantly lower levels of mental health than those who do not, and Internet use can significantly improve the mental health of older adults. The three dimensions of Internet use also have different effects on the life satisfaction of older adults, and only the use of the financial management function has a significant negative effect on life satisfaction.

**Table 4:** Robustness tests of the influence of living arrangements on the mental health of older adults and the moderating effect of Internet use.

	Model 5	Model 6	Model 7
Living arrangements	−0.140**	−0.141**	−0.142**
	(0.06129)	(0.061)	(0.061)
Internet		0.488**	
		(0.214)	
Available Internet connections			2.044
			(1.483)
Frequency of Internet use			−0.551
			(0.496)
Chatting			−0.179
			(0.442)
Entertainment			−0.374
			(0.504)
Information acquisition			0.728
			(0.466)
Financial management			−1.616***
			(0.580)
Control variable	Yes	Yes	Yes
Constant	3.929	3.697	4.164
	(2.873)	(2.872)	(2.909)
*N*	17,243	17,243	17,243
Pseudo R^2^	0.0592	0.0600	0.0610

(1) Standard errors in parentheses; (2) * *p* < 0.1, ** *p* < 0.05, *** *p* < 0.01.

### 3.3 Heterogeneity analysis

In the previous study, it was found that older adults who were male, single, unhealthy, poorly educated, with low income, and living in rural areas obtained a significantly higher depression index score. As education level, total income, and place of residence reflect the social structural differences, we chose these three variables to carry out the heterogeneity analysis (Tables 5, 6). It can be seen that the heterogeneity analyses of each group passed the Chow test, indicating that the differences between the groups were significant.

**Table 5:** Influence of living arrangements on the mental health of older adults with different levels of education.

	Model 8	Model 9	Model 10	Model 11
	Illiterate	Elementary school and below (literate)	Middle school	High school and above
Living arrangements	0.547***	0.320**	0.440**	0.707***
	(0.171)	(0.135)	(0.179)	(0.214)
Available Internet connections	1.863	−0.791	−1.210	−0.572
	(4.163)	(1.761)	(1.497)	(1.168)
Frequency of Internet use	0.597	0.415	0.038	0.253
	(1.633)	(0.575)	(0.480)	(0.375)
Chatting	−5.478*	−0.481	1.255**	−0.620
	(2.893)	(0.702)	(0.498)	(0.404)
Entertainment	−1.832	0.884	−0.222	0.276
	(3.173)	(0.757)	(0.548)	(0.473)
Information acquisition	0.699	−2.250***	−0.736	−0.985
	(2.834)	(0.774)	(0.687)	(0.619)
Financial management		0.577	0.849	1.548**
		(2.272)	(1.002)	(0.668)
Control variable	Yes	Yes	Yes	Yes
Constant	−5.091	16.272**	26.205***	31.224***
	(7.834)	(7.112)	(9.972)	(10.450)
*p value*	0.000***			
*N*	5,527	6,810	2,989	1,917
R^2^	0.074	0.099	0.105	0.135

(1) Standard errors in parentheses; (2) * *p* < 0.1, ** *p* < 0.05, *** *p* < 0.01.

**Table 6:** Influence of living arrangements on the mental health of older adults with different levels of income and place of residence.

	Model 12	Model 13	Model 14	Model 15
	Low income	High income	Rural	Urban
Living arrangements	0.452***	0.400	0.409***	0.544***
	(0.091)	(0.243)	(0.102)	(0.153)
Available Internet connections	−1.259	−1.054	0.043	−1.700
	(0.945)	(2.330)	(1.352)	(1.087)
Frequency of Internet use	0.339	−0.402	0.145	0.258
	(0.310)	(0.753)	(0.446)	(0.354)
Chatting	−0.221	0.527	0.224	−0.214
	(0.328)	(1.233)	(0.619)	(0.347)
Entertainment	0.005	2.323	−0.012	0.499
	(0.369)	(1.488)	(0.673)	(0.394)
Information acquisition	−0.950**	−1.261	−2.403***	−0.100
	(0.431)	(1.568)	(0.720)	(0.474)
Financial management	1.070*	0.922	−0.617	1.362**
	(0.615)	(3.954)	(1.889)	(0.601)
Control variable	Yes	Yes	Yes	Yes
Constant	5.627	23.660**	6.223	18.034**
	(4.777)	(10.848)	(5.158)	(7.119)
*P-value*	0.0131**		0.000***	
*N*	14,910	2,333	12,566	4,677
R^2^	0.129	0.108	0.108	0.133

(1) Standard errors in parentheses; (2) * *p* < 0.1, ** *p* < 0.05, *** *p* < 0.01.

Table 5 shows the relationship between living arrangements, Internet use, and mental health of older adults with different levels of education, and it can be seen that there were significant differences between the four groups (Chow test: *p*-value<0.001). For all four groups, older adults who do not live with their children had significantly higher levels of depression than those who live with their children. However, different dimensions of Internet use had different effects on the mental health of older adults with different levels of education. For the illiterate group, chatting significantly reduced the level of depression in older adults. However, for the middle school group, chatting significantly increased the level of depression in older adults. For the other two groups (elementary school and below (literate), high school, and above), chatting had no significant effect on the level of depression in older adults. Except for the illiterate group, information acquisition significantly reduced the level of depression in older adults in the other three groups, especially for the elementary school and below (literate) group (*p* < 0.01). Meanwhile, financial management increased the level of depression in older adults, especially in the high school and above group (*p* < 0.05).

Table 6 shows the relationship between living arrangements, Internet use, and mental health of older adults in the low-income and high-income groups and in urban and rural areas. The results showed that there was a significant difference between the low-income group and the high-income group (Chow test: *p*-value<0.05). The living arrangements of older adults in the low-income group had a significant impact on their mental health. The depression level of older adults who do not live with their children was significantly higher than that of older adults who live with their children. However, the living arrangements of older adults in the high-income group had no significant effect on their mental health. In terms of Internet use, information acquisition and financial management had a significant impact on the mental health of older adults in the low-income group. Information acquisition significantly reduced the depression level of older adults in the low-income group, but financial management significantly increased the depression level of older adults in the low-income group. For older adults in the high-income group, all dimensions of Internet use had no significant impact on their mental health.

The difference between urban and rural groups was equally significant (Chow test *p*-value<0.001). Regardless of urban and rural areas, the living arrangements of older adults had a significant impact on their mental health, and living with their children significantly reduced the level of depression in older adults. However, the impact of Internet use differed between the two groups. For rural older adults, information acquisition significantly reduced their depression levels (*p* < 0.01), while for urban older adults, information acquisition had no significant effect on their depression levels. For urban older adults, financial management significantly increased their depression levels, while for rural older adults, financial management had no significant impact on their depression levels. According to the regression coefficient, financial management also reduced the level of depression of rural older adults.

### 3.4 Moderating effect test

Table 7 verifies the moderating mechanisms of Internet use in the influence of living arrangements on the mental health of older adults (Model 16) and the specific moderating mechanisms of the three main dimensions of Internet use (Model 17). Model 16 shows that living arrangements significantly affect the mental health of older adults. Compared to older adults who live with their children, those who do not live with their children have significantly higher levels of depression. Internet use had a significant impact on the mental health of older adults, and it significantly reduced the level of depression among older adults. However, the Living*Internet interaction term did not pass the significance test, indicating that Internet use did not play a moderating role. According to the regression coefficient, Internet use weakened the influence of living arrangements on the mental health of older adults.

**Table 7:** Test of the moderating mechanism of Internet use for the influence of living arrangements on the mental health of older adults.

	Model 16	Model 17
Living arrangement	0.454***	0.535***
	(0.088)	(0.147)
Internet use	−0.859***	
	(0.219)	
Living*Internet	−0.143	
	(0.315)	
Available Internet connections		−0.986
		(1.139)
Frequency of Internet use		0.193
		(0.372)
Chatting		0.175
		(0.413)
Entertainment		−0.605
		(0.484)
Information acquisition		−0.021
		(0.507)
Financial management		−0.549
		(0.915)
Living*Available Internet connections		−0.698
		(1.732)
Living*Frequency of Internet use		0.393
		(0.571)
Living*Chatting		−1.064*
		(0.630)
Living*Entertainment		1.648**
		(0.707)
Living*Information acquisition		−1.557*
		(0.873)
Living*Financial management		3.126**
		(1.214)
Control variable	Yes	Yes
Constant	11.106***	10.918***
	(4.092)	(4.106)
*N*	17,457	17,457
Adj. R^2^	0.1289	0.1296

(1) Standard errors in parentheses; (2) * *p* < 0.1, ** *p* < 0.05, *** *p* < 0.01.

Model 17 further verifies which elements of Internet use play a moderating role in the process of living arrangements affecting the mental health of older adults and how they play a moderating role. It was found that available Internet connections and frequency of Internet use did not play a significant moderating role in the process of living arrangements affecting the mental health of older adults. In terms of the coefficient of the interaction term, available Internet connections weakened the effect of living arrangements, while the frequency of Internet use strengthened the effect of living arrangements. Meanwhile, according to Model 17, it was found that all patterns of Internet use significantly moderated the effect of living arrangements on the mental health of older adults. Among them, chatting and information acquisition significantly weakened the effect of living arrangements (*p* < 0.1), indicating that chatting and information acquisition through the Internet reduced the level of depression among older adults who do not live with their children. However, entertainment and financial management significantly enhanced the effect of living arrangements (*p* < 0.05), suggesting that entertainment and financial management through the Internet strengthened the level of depression among older adults who do not live with their children. This may be because chatting and information acquisition through the Internet can strengthen bidirectional and multidimensional communication between older adults and the outside world, while entertainment and financial management through the Internet are more likely to substitute for the social needs of older adults in the real world and enhance unidirectional communication toward the virtual world. Model 17 partially verifies Hypothesis 3, which supports the existence of a moderating effect of Internet use. However, Model 17 also demonstrates the complexity of the moderating mechanism, with inconsistent directions of moderation. Specifically, some patterns of Internet use have an enhancing effect, while others have a weakening effect, and there are two dimensions (available Internet connections and frequency of Internet use) that do not exhibit any moderating effect.

## 4 Discussion

The following revelations can be drawn from the results of the experiment.

The living arrangements of older adults have a significant impact on their mental health. Compared to older adults who live with their children, those who do not live with their children have significantly higher levels of depression, which is consistent with results of previous studies (10). As older adults are more dependent on social support, living alone may lead to insufficient interaction between older adults and their family and friends, which can lead to a greater risk of depression (31). Moreover, living alone can also increase the risk of poverty among older adults (32), thus increasing the psychological stress of older adults. At the same time, as the family remains the main source of support for older adults in China (33), Living with children can not only promote communication between older adults and their children (34) but also give older adults a sense of pride in Chinese cultural while helping older adults to obtain instrumental and emotional support from their children (35), thus maintaining the mental health of older adults.

Internet use has a significant effect on the mental health of older adults. Similar to previous studies, we confirmed that Internet use can significantly promote mental health for older adults (21, 24). This is because the Internet can provide with older adults all kinds of information, various forms of entertainment opportunities, and convenient and fast social interactions (36). Internet use not only helps older adults interact with their family but also helps them keep up with current events and access entertainment (37). Above all, the social functions of the Internet have the most obvious positive effect on mental health. Older adults can promote their mental health by communicating with family and friends through email (38) and online chat.

There are significant differences in the relationship between living arrangements, Internet use, and the mental health of older adults across different groups (education level, income, and urban and rural areas). In particular, the degree and direction of the impact of Internet use vary among different groups. Internet use plays a moderating role in the impact of living arrangements on the mental health of older adults, but it is necessary to look specifically at the moderating mechanisms for different dimensions of Internet use. In general, the social attributes of the Internet (chatting and information acquisition) can weaken the impact of living arrangements on the mental health of older adults, while unidirectional Internet use (entertainment and financial management) strengthens the impact of living arrangements on the mental health of older adults. At the same time, whether older adults are connected to the Internet and the frequency of Internet use does not have a moderating effect in the impact of living arrangements on the mental health of older adults. The reason is that Internet use can enhance communication between older adults and their children (39) and help older adults maintain a close intergenerational relationship (40). At the same time, Internet use also helps older adults meet new friends (41) and expand their social network, thus maintaining close contact with society (42).

The study still has some limitations. First, as the data used in this study were collected in 2018, this study does not reflect the situation at present. Second, although we have used five control variables (age, sex, education level, physical health, and place of residence), it is still difficult to rule out possible conflations caused by factors such as socioeconomic level. Furthermore, this study revealed the complex mechanisms by which Internet use moderates the impact of living arrangements on the mental health of older adults. The measurement of Internet use was multidimensional, and its moderating mechanisms exhibited both compensatory and substitutive effects. This paper only investigated Internet use as a moderating variable and did not explore how Internet use affects the mental health of older adults and its underlying mechanisms. However, based on the existing findings, we believe that it is necessary to conduct dedicated research on the mechanisms by which Internet use influences the mental health of older adults, including whether these mechanisms change with changes in variables such as age. This will be the direction for future research.

## 5 Conclusion

This paper concludes that older adults living with their children have better mental health than those living independently. In addition, Internet use is beneficial in promoting mental health among older adults and the relationship between living arrangements, Internet use, and mental health is evident. Finally, this study supports the moderating effect of Internet use, which has policy implications for improving the mental health of older adults in the digital age. However, the mechanism of the role of the Internet should be analyzed specifically and cannot be discussed in general terms.

Living arrangements reflect intergenerational support to a certain extent, and living with children can significantly reduce the level of depression of older adults. This indicates that the spiritual comfort function of family care is still very prominent. Even in the digital age, family care for older adults is still very important. Therefore, the government can encourage older adults to live with their children in order to improve their mental health. If it is really impossible to live together, the government should encourage the children of older adults to use the Internet to strengthen their care for older adults and provide them with the necessary emotional support.

At the same time, the government can integrate the strength of families and peer groups to provide targeted digital education for older adults, guide them to screen Internet content effectively, help them develop good habits of Internet use, enhance their ability to use the Internet, so as to help older adults to enjoy the benefits of Internet use as much as possible.

On the one hand, it is necessary to reasonably guide older adults to use the Internet to expand their social communication (chatting and obtaining information). Utilizing the Internet to expand the connections between older adults and the real-world society is feasible because Internet use transcends spatial constraints and enables children who do not live with their parents to provide support through the Internet. This can partly diminish the need for older adults to rely on face-to-face support from their children. On the other hand, it is important to be aware of the potential risks associated with Internet use. While the Internet can provide necessary support for older adults, it may also expose them to the risk of becoming immersed in the virtual world through activities such as watching videos, playing online games, and financial management online. Consequently, this may lead to the impairment of their mental health.

Therefore, the government and social service departments must assist older adults in establishing correct concepts of Internet use, enhancing their digital literacy, and improving their digital skills.

## Data Availability

The original contributions presented in the study are included in the article/supplementary material, further inquiries can be directed to the corresponding author.
